# Visual Feature-Guided Diamond Convolutional Network for Finger Vein Recognition

**DOI:** 10.3390/s24186097

**Published:** 2024-09-20

**Authors:** Qiong Yao, Dan Song, Xiang Xu, Kun Zou

**Affiliations:** Artificial Intelligence and Computer Vision Laboratory, Zhongshan Institute, University of Electronic Science and Technology of China, Zhongshan 528402, China; yaoqiong@zsc.edu.cn (Q.Y.);

**Keywords:** finger vein, Log-Gabor, diamond convolutional network, visual feature-guided

## Abstract

Finger vein (FV) biometrics have garnered considerable attention due to their inherent non-contact nature and high security, exhibiting tremendous potential in identity authentication and beyond. Nevertheless, challenges pertaining to the scarcity of training data and inconsistent image quality continue to impede the effectiveness of finger vein recognition (FVR) systems. To tackle these challenges, we introduce the visual feature-guided diamond convolutional network (dubbed ‘VF-DCN’), a uniquely configured multi-scale and multi-orientation convolutional neural network. The VF-DCN showcases three pivotal innovations: Firstly, it meticulously tunes the convolutional kernels through multi-scale Log-Gabor filters. Secondly, it implements a distinctive diamond-shaped convolutional kernel architecture inspired by human visual perception. This design intelligently allocates more orientational filters to medium scales, which inherently carry richer information. In contrast, at extreme scales, the use of orientational filters is minimized to simulate the natural blurring of objects at extreme focal lengths. Thirdly, the network boasts a deliberate three-layer configuration and fully unsupervised training process, prioritizing simplicity and optimal performance. Extensive experiments are conducted on four FV databases, including MMCBNU_6000, FV_USM, HKPU, and ZSC_FV. The experimental results reveal that VF-DCN achieves remarkable improvement with equal error rates (EERs) of 0.17%, 0.19%, 2.11%, and 0.65%, respectively, and Accuracy Rates (ACC) of 100%, 99.97%, 98.92%, and 99.36%, respectively. These results indicate that, compared with some existing FVR approaches, the proposed VF-DCN not only achieves notable recognition accuracy but also shows fewer number of parameters and lower model complexity. Moreover, VF-DCN exhibits superior robustness across diverse FV databases.

## 1. Introduction

Finger vein (FV) biometrics has emerged as an exceptionally secure and reliable technology for personal identity authentication. Finger veins are vascular pattern features that are imperceptible to the naked eye, but can be captured by using near-infrared (NIR) light with a specific wavelength ranging from 700 nm to 1000 nm [1]. When NIR light passes through the finger, blood vessels absorb the light, causing a distinctive dark pattern on the image. Such unique vein patterns offer several advantages over other biometric traits, including:

- Highsecurity. The intricate and distinctive patterns of FV are unique, rendering them exceedingly difficult to replicate or forge.
- Non-contact. Finger vein recognition (FVR) does not require physical contact with the sensor, significantly reducing the risk of contamination and the transmission of germs.
- User-friendly. The process of FVR is swift and straightforward, simply requiring the user to put their finger close to the sensor. Moreover, FVR is accessible to a wide range of individuals, regardless of age, gender, or complexion.

The cornerstone of FVR lies in the extraction of discriminative features from acquired images, which can be achieved through two types of primary methods: the handcrafted-based and the deep learning-driven. In the early stages of research, Miura [2,3] pioneered curvature-based methods that captured the extent of curve bending at a particular point, albeit being susceptible to noise. Later, Gabor filtering-based methods [4,5] were introduced to enhance and extract FV features, while Gabor filters are tunable to detect specific frequencies and orientations, finding optimal parameters for a given dataset remains challenging. Subsequently, curvature and Radon-like features (RLFs) were combined to effectively aggregate spatial information around vein structures [6], highlighting vein patterns and suppressing spurious non-boundary responses and noise. However, the obtained features are influenced by illumination variations. Recently, binary patterns of phase congruency (BPPCs) and pyramids of histograms of orientation gradients (PHOGs) have been incorporated for FV feature extraction [7]. However, this method remains susceptible to local changes in scale, translation, and other factors. Handcrafted-based methods rely heavily on expert experience rather than data-driven, which are not always efficient but tend to vary across databases and scenarios.

On the contrary, deep learning-driven methods, which are inherently reliant on training data, have the potential to address some of these challenges. Various classical convolutional neural networks (CNNs), such as VGGNet [8,9], AlexNet [10,11], ResNet [12], DenseNet [13,14,15], Siamese Networks [16,17], Xception [18], and generative adversarial networks (GANs) [19], have demonstrated robustness in a range of image recognition issues, and also exhibited outstanding performance in FVR through fine-tuning and transfer learning [20]. In addition, self-attention mechanisms are also explored in FVR. Among them, a vein pattern constrained transformer (VPCFormer) [21] was proposed that incorporates a self-attention mechanism to capture the correlations between different views of FV patterns, helping the model learn more discriminative features and improving its robustness. Then, a large kernel and attention mechanism network (Let-Net) [22] was presented that also utilizes a self-attention mechanism to enhance the feature representation. By incorporating large kernels and an attention mechanism, the network can capture both local and global context information. SE-DenseNet-HP [23], on the other hand, combined the squeeze-and-excitation (SE) channel attention with a hybrid pooling mechanism, allowing the model to dynamically recalibrate channel-wise feature responses and extract discriminative multi-scale features. The attention mechanism acquires the attention weights by calculating the similarity between different units (channels and channels, pixels and pixels) in the feature maps, thus achieving a concentration of information.

It is noteworthy that the attention mechanism typically elevates the computational and storage requirements of the network, necessitating longer training and inference times. In certain scenarios, the attention mechanism might inadvertently concentrate on irrelevant features, potentially causing the model to overlook crucial information [24]. In contrast, the human visual system possesses a swift and dynamic ability to adjust its perception of external objects. When the visual range is optimally positioned, it can effortlessly capture intricate details. Conversely, for objects situated too far or too close, the visual system instinctively lowers its resolution to prioritize discernible features, given the challenges of distinguishing finer details.

To address these challenges and harness the strengths of both traditional visually guided handcrafted methods and deep learning (DL) methods, while minimizing their respective limitations, we propose a uniquely configured multi-scale and multi-orientation convolutional neural network. This unique architecture, coined the visual feature-guided diamond convolutional network (hereinafter dubbed ‘VF-DCN’), boasts a deliberate three-layer configuration and fully unsupervised training process, focusing on attaining simplicity and optimal performance. In all convolutional layers of VF-DCN, the convolutional kernels are tuned through multi-scale Log-Gabor filters, and then, an adaptive orientational filter learning strategy for the convolutional kernels across different scales is implemented that draws on the human vision. Remarkably, VF-DCN showcases an innovative diamond-shaped convolutional structure that efficiently maintains a wider range of orientational kernels at medium scales. The main contributions of this work are summarized as follows:

- Visual feature-guided convolutional kernels. The Log-Gabor filters, which closely mimic the frequency response of visual cells, are used to generate multi-scale Log-Gabor convolutional kernels. This ingenious design empowers the network to capture visual features with unprecedented effectiveness.
- Diamondconvolutionalstructure. Inspired by retina imaging, where images become blurred at extreme focal lengths, a diamond convolutional structure is crafted to extract significant orientational information through training across multi-scale Log-Gabor filters.
- Fullyunsupervisedlearningnetwork. The network is deliberately designed with just two Log-Gabor convolutional layers and a fully unsupervised training process, achieving a harmonious balance between simplicity and efficiency.

The remainder of this paper is organized as follows: Section 2 provides a summary review of Gabor and Log-Gabor filtering approaches for FVR. Section 3 details the design of Log-Gabor convolutional kernels. Section 4 elaborates on the entire recognition process of the proposed VF-DCN model. Section 5 discusses the experimental results to comprehensively assess the performance of the VF-DCN model. Four FV databases are adopted that contain images with varying qualities, resolutions, and dynamic ranges. Section 6 concludes the work with some remarks and hints at plausible future research lines.

## 2. Related Works

In this section, we provide a concise overview of Gabor-like filters, specifically Gabor and Log-Gabor, in the context of FVR applications. The Gabor filter family, inspired by the receptive fields of simple cells in the mammalian visual cortex, exhibits robustness to distortion in their coefficient magnitudes, rendering them ideally suited for pattern recognition tasks [25], including those pertaining to finger veins.

### 2.1. Gabor Filters

In the field of FVR, Gabor filters have been broadly used for feature enhancement and representation. Among them, a bank of even-symmetric Gabor filters with 8 orientations was used to exploit vein information in the images [4]. Then, Yang et al. [26] extended the Gabor filter bank to 2 scales and 8 orientations, and Wang et al. [27] used a bank of 24 Gabor filters covering 4 scales and 6 orientations. Moreover, fusion schemes are introduced to offer insight into the complementarity of various feature extraction methods. Specifically, a fuzzy-based fusion method was proposed in [28] that integrated Gabor filters with Retinex filters, resulting in enhanced visibility and recognition capabilities for FV images. In [29], adaptive Gabor filters were combined with SIFT/SURF feature extractors to enhance vein patterns. In [30], the concept of point grouping was incorporated into Gabor filters to effectively capture local vein patterns. The above Gabor filtering technologies primarily extract texture and orientation features in FV images, which are susceptible to image blurring, translation, rotation, and noise. To address these issues, Shi et al. [31] incorporated scattering removal techniques with Gabor filters to improve the clarity and reliability of FV patterns, alleviating the interference of noise and blurring artifacts. Li et al. [32] proposed a histogram of competitive Gabor directional binary statistics (HCGDBS) approach to improve the discriminant ability of features and robustness to variation in image quality.

In recent years, numerous efforts have been directed towards integrating Gabor filters with deep learning networks, aimed at eliminating the constraints of manual parameter tuning and the limited representation capacity of Gabor filters. In [33], Gabor filters were employed as a preprocessing step, where Gabor-filtered images served as the input of the network. Further, in [34], the first layer of the network used Gabor kernels for feature learning, leaving the rest of the layers unchanged. Notably, the parameters of Gabor kernels are learned by backpropagation. In [35], a few of the early convolutional layers were substituted by a parameterized Gabor convolutional layer. Moreover, Luan et al. [36] adopted Gabor filters to modulate learnable convolutional kernels, allowing the network to capture more robust features across orientation and scale variations, without incurring additional computational burden. Similarly, Yao et al. [17] introduced Gabor orientation filters (GoFs) to modulate conventional convolutional kernels and constructed a Siamese network for FV verification.

It is crucial to acknowledge that Gabor filters possess two prominent limitations. First, the maximum bandwidth of a Gabor filter is constrained to approximately one octave, which restricts its ability to cover a wide range of frequencies. Second, Gabor filters are not the preferred choice when seeking broad spectrum information while requiring optimal spatial localization, as this hinders their efficiency in FV feature extraction.

### 2.2. Log-Gabor Filters

The Log-Gabor filter, proposed by Field [25], serves as an alternative to the Gabor filter with several distinct advantages. In the frequency domain, the Log-Gabor filter exhibits an attenuation rate that aligns more closely with the human visual system. This characteristic makes it more sensitive to low-frequency information and less sensitive to high-frequency information. As a result, the Log-Gabor filter demonstrates stronger anti-interference ability and is more accurate and reliable in extracting multi-scale image features. Among them, Gao [37] pioneered the use of Log-Gabor filters to decompose input images into multiple scales and orientations. Arrospide [38] demonstrated the superiority of Log-Gabor filters over Gabor filters in the context of image-based vehicle verification. Yang et al. [39] employed phase congruency and Log-Gabor energy for multimodal medical image fusion, showcasing the filters’ versatility in fusing diverse image modalities. Bounneche [40] proposed an oriented multi-scale Log-Gabor filter tailored for multispectral palmprint recognition. Lv et al. [41] utilized an odd-symmetric 2D Log-Gabor filter to analyze the phase and amplitude of iris textures across different frequencies and orientations. Shams et al. [42] combined a diffusion-coherence filter with a 2D Log-Gabor filter to enhance fingerprint images. Beyond these applications, Log-Gabor filters have also found their niche in motion estimation [43], remote sensing [44], and numerous other domains.

Overall, Log-Gabor filters exhibit superior performance compared to Gabor filters across various image processing and computer vision applications, particularly in multi-scale feature extraction, frequency feature matching, and noise resilience. Given that Log-Gabor has not yet been harnessed in FVR, we propose to incorporate Log-Gabor filters into the design of a lightweight FVR network. In the following, we will delve into the formulation of Log-Gabor convolutional kernels and the recognition process of our proposed VF-DCN model.

## 3. Log-Gabor Convolutional Kernels

In this section, 1D and 2D Log-Gabor filtering kernels are presented, and their corresponding parameter selection is discussed.

### 3.1. Log-Gabor Function

As described in [25], the transfer function of a Log-Gabor filter is a Gaussian function on a logarithmic frequency scale, the corresponding 1D Log-Gabor function is defined as Equation (1):

(1) $$\mathrm{logG}\left(f\right)=\exp\left(-\frac{{\left[\log\left(\frac{f}{f_{0}}\right)\right]}^{2}}{2{\left[\log\left(\frac{\sigma }{f_{0}}\right)\right]}^{2}}\right),$$

where $f_{0}$ is the central frequency of the filter, and σ is the standard deviation used to determine the filter bandwidth. It can be observed from Equation (1) that the frequency response of a Log-Gabor is symmetric on a logarithmic axis.

When extending the 1D Log-Gabor filter to 2D, the filter *f* in Equation (1) should be constructed in the polar coordinate system of frequency domain due to the singularity of log function at the origin. Specifically, the 2D Log-Gabor is decomposed into two components: radialfilter and angularfilter, so that the bandwidth of each component can be adjusted independently to facilitate analysis. Among these, the radial filter provides a frequency response to determine the frequency band, as described by Equation (2):

(2) $${\mathrm{logG}}_{r}\left(r\right)=\exp\left(-\frac{{\left[\log\left(\frac{r}{f_{0}}\right)\right]}^{2}}{2\cdot \sigma_{r}^{2}}\right),$$

and the angular filter is used to determine the orientation, as described by Equation (3):

(3) $${\mathrm{logG}}_{\theta }\left(\theta \right)=\exp\left(-\frac{{\left(\theta -\theta_{0}\right)}^{2}}{2\cdot \sigma_{\theta }^{2}}\right).$$

Then, these two components are multiplied together to construct the overall 2D Log-Gabor filter, as shown in Equation (4):

(4) $$\mathrm{logG}\left(r,\theta \right)={\mathrm{logG}}_{r}\left(r\right)\cdot {\mathrm{logG}}_{\theta }\left(\theta \right),$$

where (r,θ) are the polar coordinates, with *r* representing the radial coordinate and θ representing the angular coordinate. $\theta_{0}$ is the orientation angle of the filter, and $\sigma_{r}$ and $\sigma_{\theta }$ are used to determine the radial and angular bandwidths, respectively. Table 1 shows the parameter settings required to build the two components of the 2D Log-Gabor filter, and the selection of specific parameters is discussed below.

### 3.2. Radial Parameters Selection

**Figure 1:**
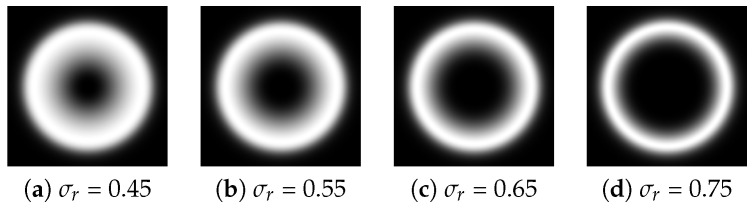
Radial filters under different values of $\sigma_{r}$.

In Equation (2), $\sigma_{r}$ determines the radial filter bandwidth. The smaller the value of $\sigma_{r}$, the larger the radial filter bandwidth. Empirically, when the value of $\sigma_{r}$ is 0.75, the radial filter bandwidth is approximately one octave, and when the value of $\sigma_{r}$ is 0.55, the radial filter bandwidth is approximately two octaves. Figure 1 shows the results of the radial filters under different values of $\sigma_{r}$. In our experiments, $\sigma_{r}$ is set to 0.55 for balancing purposes.

In addition, the filter’s central frequency $f_{0}$ is calculated by Equation (5) as the reciprocal of the wavelength.

(5) $$f_{0}=\frac{1}{wavelength}.$$

Here, the wavelength is calculated by Equation (6).

(6) $$wavelength=W_{min}\cdot M^{S-1},\;\left(S=1,\ldots ,N_{scale}\right).$$

$W_{min}$ is the wavelength of the smallest scale filter, *M* is the radial scaling factor, which is used to control the successive wavelength of the radial filters, and *S* denotes the radial filter scales varying from 1 to $N_{scale}$. When the wavelength is set to the minimum value $W_{min}$, the frequency attains its maximum value. In Section 5.4.1, we discussed the influence of different $W_{min}$ on the recognition performance and set $W_{min}=2$ pixels.

### 3.3. Angular Parameters Selection

In Equation (3), $\theta_{0}$ is the orientation angle of the filter, as defined by Equation (7):

(7) $$\theta_{0}=i\cdot \frac{\pi }{N_{ori}},\;\left(i=0,\ldots ,N_{ori}-1\right).$$

Similarly, the angular bandwidth of the filter is determined by the parameter $\sigma_{\theta }$, which is calculated by Equation (8).

(8) $$\sigma_{\theta }=T\cdot \frac{\pi }{N_{ori}}.$$

**Figure 2:**
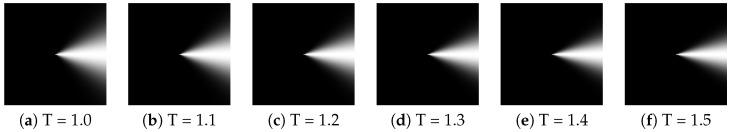
Angular filters under different angular scaling factors *T*.

The angular bandwidth determines the directionality of the filter, a narrower bandwidth results in stronger directionality. Moreover, the angular interval between filter orientations is fixed by $N_{ori}$. In the frequency domain, the spread of the 2D Log-Gabor filter in the angular orientation is a Gaussian with respect to the polar angle around the center. The angular overlap of the filter transfer functions is controlled by the angular interval between filter orientations and angular scaling factor *T*. Figure 2 shows the results of angular filters when $\theta_{0}=0$, $N_{ori}=10$ under different angular scaling factors *T*. It can be observed that the larger the values of *T*, the less angular overlap. In the following experiments, *T* is set to 1.3 to achieve approximately minimal overlap.

### 3.4. Bank of Log-Gabor Filtering Kernels

**Figure 3:**
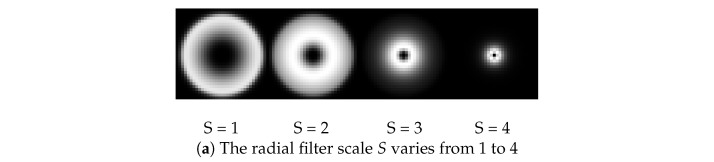

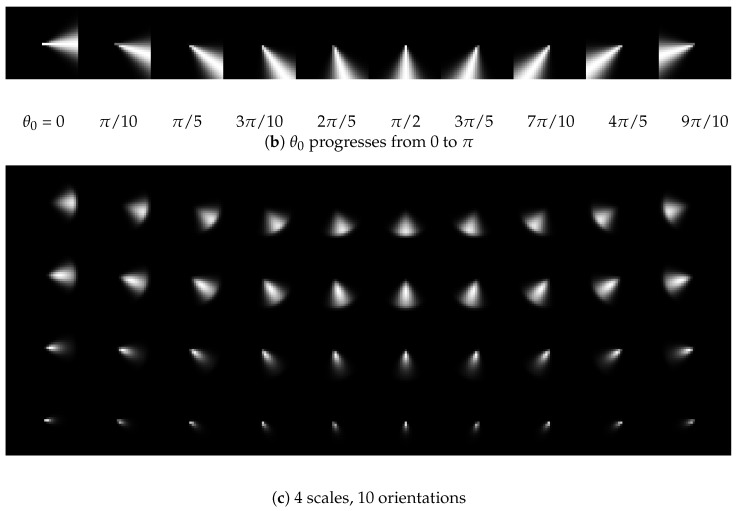
Bank of Log-Gabor filters. Each row in (**c**) contains filters computed with the same scale, for each scale, 10 orientations are sampled.

With the parameter settings of $N_{scale}=4$ and $N_{ori}=10$, we can obtain a bank of 2D Log-Gabor filters by Equation (4). According to the parameter settings in Table 1, we present the bank of Log-Gabor filters obtained in Figure 3.

## 4. VF-DCN Model for Finger Vein Recognition

As previously discussed, the human visual system exhibits nonlinear logarithmic characteristics. In this regard, Log-Gabor is consistent with the human visual system, potentially enabling it to encode natural images more efficiently than ordinary Gabor functions. Given the remarkable performance gains achieved by Gabor filters integrated with CNNs in the field of FVR, it is reasonable to hypothesize that the incorporation of Log-Gabor filters into CNNs could further bring improvements. Motivated by the above premise, we integrated Log-Gabor filters with a CNN architecture to devise a uniquely configured multi-scale and multi-orientational finger vein recognition network, namely ‘VF-DCN’.

In this section, the overall framework of our VF-DCN and its processing flow specific to FVR are firstly elaborated. Then, an adaptive orientational filter selection and retention mechanism for Log-Gabor convolutional kernels across various scales is implemented. This stands as the cornerstone of our VF-DCN model, ensuring optimal utilization of Log-Gabor filters for capturing intricate vein patterns across different orientations and scales. Finally, the output feature vectors of image samples are extracted from the well-trained VF-DCN and serve as inputs for downstream recognition or verification tasks.

### 4.1. Framework of VF-DCN Model

**Figure 4:**
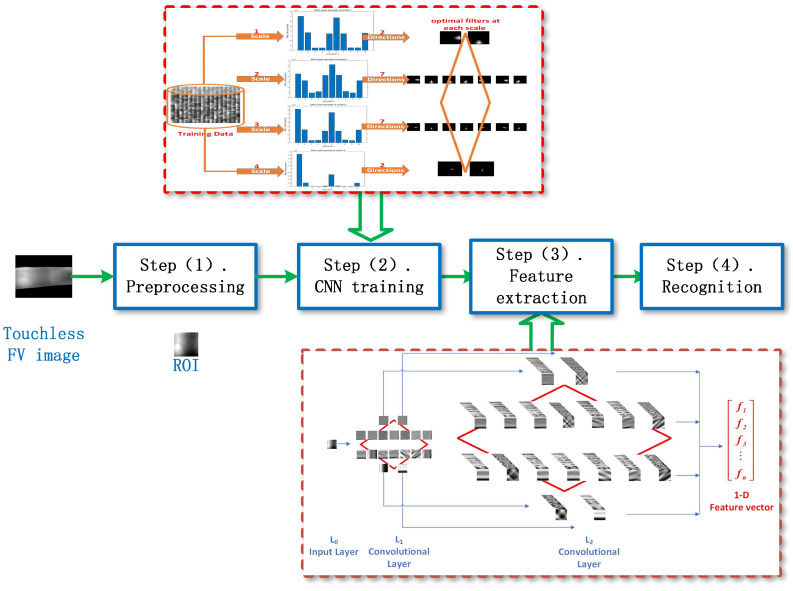
Illustration of the framework of VF-DCN.

The overall framework of the VF-DCN is depicted in Figure 4. It is known as a lightweight network, consisting of a preprocessing stage and an unsupervised training process. Here, the unsupervised training aims to learn the convolutional kernels within its two convolutional layers. By utilizing multi-scale Log-Gabor filters and incorporating the human visual system’s sensitivity to orientation at varying scales, the optimal orientational filters are adaptively identified and function as the final convolutional kernels. For detailed unsupervised training strategies, refer to Section 4.2. Upon completion of the thorough training process, the VF-DCN model transforms into a feature extractor, generating feature vectors that can be directly employed in downstream recognition or verification tasks.

#### 4.1.1. Preprocessing Stage

In the preprocessing step, we employed a synergistic approach that integrates the 3σ criterion dynamic threshold strategy [1] with the Kirsch detector [45] to localize the region of interest (ROI). Compared to Sobel, Canny, etc., the Kirsch detector exhibits a superior balance in identifying weak edges and minimizing false edges, yielding a clearer binary edge gradient image. Nonetheless, when FV image quality is hindered by uneven illumination and noise, edges may exhibit pronounced discontinuities, and some weak edges may remain undetected. To address this issue, the 3σ criterion offers three-level dynamic thresholds that automatically adjust to varying image qualities. This ensures the generation of more complete boundary lines, thereby facilitating the efficacy of the ROI extraction process. For illustration, Figure 7c,d show examples of ROI extracted from two FV databases.

#### 4.1.2. Unsupervised Training Process of VF-DCN

In this section, we initially illustrate the network topology of VF-DCN, followed by a detailed exposition of its specific training process.

**Figure 5:**
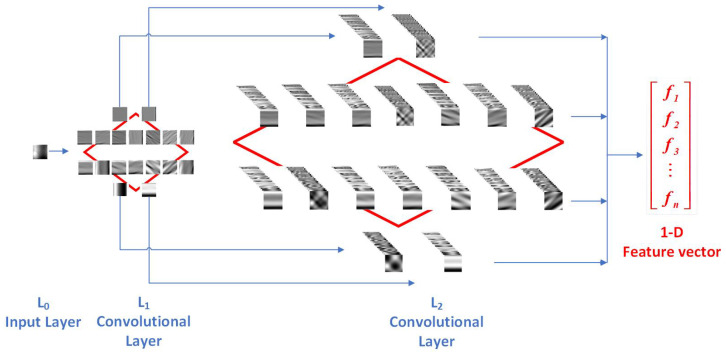
Diamond convolutional structure of VF-DCN.

The backbone of VF-DCN boasts a deliberate three-layer CNN architecture consisting of two consecutive Log-Gabor convolutional layers ($L_{1}$ and $L_{2}$), followed by a binary hashing and block-wise histogram layer. As shown in Figure 5.

The input layer $L_{0}$ comprises the ROI samples derived from the preprocessing stage. Assuming the *i*-th input ROI sample $I_{i}$ possesses dimensions of μ×ν. For two consecutive Log-Gabor convolutional layers, $N_{scale}$ scales and $N_{ori}$ orientations of Log-Gabor filters are adaptively constructed, comprising a bank of $K_{1}$ and $K_{2}$ filtering kernels in each convolutional layer. In the first convolutional layer, each of $K_{1}$ filtering kernel is convolved with the input sample $I_{i}$, forming a total of $K_{1}$ output feature maps $I_{i}^{\ell_{1}}$ with dimensions of μ×ν, as mathematically expressed in Equation (9):

(9) $$\begin{array}{c} I_{i}^{\ell_{1}}=I_{i}*{\mathrm{logG}}_{\ell_{1}}^{1},\;\ell_{1}=1,\ldots ,K_{1}, \end{array}$$

where * signifies the 2D Log-Gabor convolution operation.

After the completion of the first convolutional layer, each $K_{1}$ input feature map $I_{i}^{\ell_{1}}$ undergoes a convolution operation with every convolution kernel in ${\mathrm{logG}}^{2}$, resulting in a total of $K_{1}\times K_{2}$ output feature maps with dimensions of μ×ν. This transformation is concisely encapsulated in Equation (10):

(10) $$I_{i}^{\ell_{1},\ell_{2}}=I_{i}^{\ell_{1}}*{\mathrm{logG}}_{\ell_{2}}^{2},\;\ell_{1}=1,\ldots ,K_{1},\ell_{2}=1,\ldots ,K_{2}.$$

Subsequently, binary hashing is performed on the acquired feature maps, and the final histogram features are distilled through block-wise histogram encoding. In this process, the binary layer serves as a nonlinear transformer, leveraging a straightforward binary hashing quantization method to remap the feature maps into a binary representation, as expressed in Equation (11).

(11) $$\begin{array}{c} T_{i}^{j}=\sum_{k=1}^{K_{2}}2^{k-1}H\left(I_{i}^{j,k}\right),\;j=1,\ldots ,K_{1}, \end{array}$$

where H(·) is a Heaviside step function that outputs 1 when the variable is positive and 0 otherwise. ∑ denotes the weighted sum of $K_{2}$ binary images, so as to obtain the encoded feature maps with integer-valued mode.

The block-wise histogram layer plays the role of feature pooling. It uses simple block-wise histograms of the binary encoding to generate the final 1D feature vector. First, feature map $T_{i}^{j}$ is partitioned into B number of non-overlapping blocks. Then, the histogram of decimal values in each block is computed, and all B block histograms are concatenated into a 1D vector, as expressed in Equation (12).

(12) $$\begin{array}{c} f_{i}={\left[\mathrm{Hist}\left(T_{i}^{1,1}\right),\ldots ,\mathrm{Hist}\left(T_{i}^{1,B}\right),\ldots ,\mathrm{Hist}\left(T_{i}^{K_{1},1}\right),\ldots ,\mathrm{Hist}\left(T_{i}^{K_{1},B}\right)\right]}^{T}, \end{array}$$

where Hist(·) is the histogram operation function, and $f_{i}\in {ℜ}^{\left(2^{K_{2}}\right)K_{1}B}$ is the learned feature vector corresponding to the input image sample $I_{i}$.

In short, VF-DCN innovatively incorporates Log-Gabor convolutional kernels to extract multi-scale and multi-orientation human-like visual features, which mitigates overfitting and simplifies the training process. It can be seen as a simple unsupervised deep convolutional network, allowing for random sample selection during network training without the need to tune or optimize various regularization parameters. Moreover, the block-wise histogram of VF-DCN implicitly encodes spatial information in the image, effectively approximating the probability distribution function of image features within each block.

### 4.2. Adaptive Orientational Filtering Selection

As previously mentioned, the key training objective revolves around determining the optimal Log-Gabor convolutional kernels across two consecutive convolutional layers. To achieve this, we devised an adaptive orientational filter selection and retention strategy across multiple scales, tailored to extract multi-scale features while dynamically selecting the most suitable orientational filters for diverse FV datasets. The learning process of the adaptive filter consists of three main steps:

- Firstly, a candidate bank of Log-Gabor filters is constructed, comprising 4 scales and 10 orientations. Specifically, the radial filter scale *S* (as denoted in Equation (6)) is set to {1,2,3,4}, and the orientation angle $\theta_{0}$ (as denoted in Equation (7)) spans from {0,π/10,π/5,3π/10,2π/5,π/2,3π/5,7π/10,4π/5,9π/10}.
- Secondly, for each scale, we carry out a histogram statistical analysis of the most pertinent orientational filters. It should be noted here that the reason why each scale is carried out separately is inspired by the nature of retinal imaging, where fine details become harder to discern at extreme distances due to declined detail resolution, we should adjust to varying focal lengths and perspectives when analyzing objects at different scales. Likewise, in the convolutional layer of the VF-DCN, it becomes imperative to dynamically adjust the number of orientational filters based on the scale’s suitability in extracting features. To address this, we carry out the selection of orientational filters within each scale in turn. Specifically, aims to the aforementioned 10 candidate orientational filters within each scale, each training ROI image $I_{i}$ is convolved with them, resulting in a total of 10 filtered complex images (denoted as $resF_{ij}$, j=1,…,10). Subsequently, we extract the absolute value of the real part from each filtered complex image $resF_{ij}$ to generate the corresponding power map (denoted as $powMapF_{ij}$). Next, the magnitude responses of each pixel in these power map images serve as a metric for assessing the filter’s impact on the image. We then sort these magnitude responses in descending order across all pixels and all power maps, simultaneously recording the index of the power map, as well as the corresponding spatial row and column coordinates. This enables us to identify the most prominent orientations—those filters most frequently utilized—by analyzing the statistical histogram of high magnitude responses among the candidate orientational filters.
- Finally, we retain the filters with the highest count of such high-magnitude responses, effectively fine-tuning the number of orientations at each scale. This strategy ensures that the convolutional filters better reflect the inherent characteristics of the image and the scale’s contribution to feature extraction. By mirroring the adaptability of the human visual system in processing objects at varying distances, this mechanism enhances the efficiency and realism of the convolutional filters.

In order to better understand the whole process of orientational filtering selection, we provide a pseudo-code description in Algorithm 1.

In Algorithm 1, logGabor is the Log-Gabor filter construction function, enabling the generation of Log-Gabor filters tailored to specific scales and orientations as dictated by Formula (4). To efficiently perform approximate Log-Gabor image convolution operations, the algorithm leverages the fft2 and ifft2 functions, which represent the two-dimensional discrete Fourier transform and its inverse transform, respectively. Following the convolution operations, the real() function is employed to isolate the real part of the transformed data. The SortFilterResponse() function, whose pseudo-code is detailed in Algorithm 2, plays a pivotal role in sorting the magnitude responses of each pixel across all orientational power maps. Subsequently, the CountMostUsedOri() function, accompanied by its pseudo-code in Algorithm 3, delves into statistical analysis. It meticulously counts the frequency of occurrence of each candidate orientational filter across all pixel positions. Finally, the SelectMostUsedOri() function simplifies the process by directly identifying and selecting the most frequently used orientational filters from the pool of candidates. This streamlined approach ensures that the most representative filters are prioritized for further analysis or application.
**Algorithm 1** Pseudo-code of the orientational filter selection algorithm**Input:**  1:Training ROI images: $I_{i}$, i=(1,…,N);  2:Radial filter scale: S={1,2,3,4};  3:Candidate orientation angle of the filter: $\theta_{0}=\{0,\pi /10,\pi /5,3\pi /10,2\pi /5,\pi /2,3\pi /5,$7π/10,4π/5,9π/10};  4:Number of scales $N_{scale}=4$, number of candidate orientations $N_{ori}=10$.**Output:**  5:The best orientation angles: $best_{\theta_{0}}$.
  6:
  7:// Construct init bank of Log-Gabor filters.  8:**for** *s* = 1 to $N_{scale}$ **do**  9:    $FilterBank\left(s\right)=\mathrm{logGabor}\left(s,\theta_{0}\right)$;10:**end for**
11:
12:// Select the best orientation filters within each scale in turn.13:**for** *s* = 1 to $N_{scale}$ **do** // for each scale14:    **for** *i* = 1 to *N* **do** // for each training sample15:        **for** *j* = 1 to $N_{ori}$ **do**// for each orientation16:           $resF_{ij}=\mathrm{ifft}2\left(\mathrm{fft}2\left(I_{i}\right)\;.*\;FilterBank\left(s\right)\left(j\right)\right)$;17:           $powMapF_{ij}=\left|(\mathrm{real}\left(resF_{ij}\right))\right|$; // calculate absolution of real part.18:        **end for**19:        // Record and sort magnitude responses of each pixel in all orientational power maps.20:        $sortRes_{i}=\mathrm{SortFilterResponse}\left(powMapF_{i}\right)$;21:        // statistical histogram of the candidate orientational filters.22:        $ori\_count_{i}=\mathrm{CountMostUsedOri}\left(sortRes_{i},FilterBank\left(s\right)\right)$;23:        $ori\_countAll+=ori\_count_{i}$;24:    **end for**25:    $best_{\theta_{0}}=\mathrm{SelectMostUsedOri}\left(ori\_countAll\right)$. // Choose the most used orientational filters.26:**end for**


As illustrated in Figure 3, filters corresponding to extreme scales, specifically S = 1 and S = 4, are overly large or small, respectively. Conversely, filters at intermediate scales, notably S = 2 and S = 3, contribute more significantly to capturing crucial features. Consequently, for the extreme scales (S = 1 and S = 4), we strategically select a relatively fewer orientational filters (e.g., n1 = n4 = 2), while for the intermediate scales (S = 2 and S = 3), we retain a comparatively higher number of orientational filters (e.g., n2 = n3 = 7).

**Figure 6:**
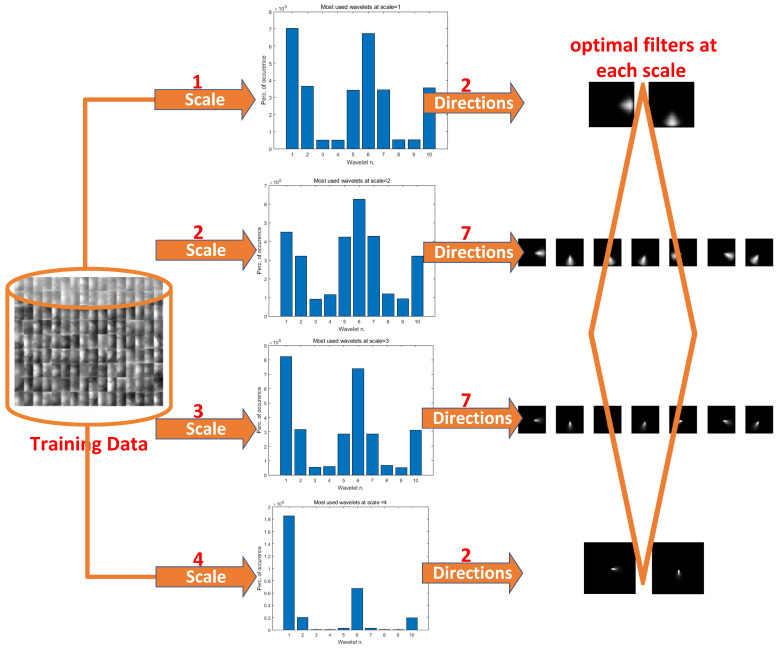
Adaptive orientational filter learning strategy for the convolutional kernels across different scales.

Surprisingly, the acquired convolutional kernel structure resembles a diamond shape, aptly modeling the human eye’s adaptability to varying focal lengths and perspectives when observing objects at different distances. This feature not only brings a bio-plausible mechanism but also significantly enhances the robustness of a computer vision model when processing real-world images. Figure 6 depicts the adaptive orientational filter learning strategy applied to the convolutional kernels across diverse scales. This strategy enables the model to dynamically refine its orientation selection, optimizing its performance based on the intricacies of the data it encounters.
**Algorithm 2** Pseudo-code for SortFilterResponse() function**Input:**  1:Num of pixels: $N_{pix}=N_{ori}\times r\times c$;  2:Power Maps: $powMapF\in {ℜ}^{N_{ori}\times r\times c}$.**Output:**  3:Sorted magnitude responses of all pixels: $sortRes\in {ℜ}^{N_{pix}\times 4}$.
  4:
  5:$sortRes=zeros(N_{pix},4)$;  6:$temp=zeros(N_{pix},4)$;  7:idx=1;  8:**for** *i* = 1 to $N_{ori}$ **do** // for each orientational power map  9:    **for** rows = 1 to *r* **do**10:        **for** cols = 1 to *c* **do**11:           tmp(idx,1)=powMapF(i).value(rows,cols);12:           tmp(idx,2)=rows;13:           tmp(idx,3)=cols;14:           tmp(idx,4)=i;15:           idx++;16:        **end for**17:    **end for**18:**end for**
19:
20:// Sort magnitude responses of each pixel in descending order.21:$\left[sortRes\left(:,1\right),idxSort\right]=sort\left(tmp\left(:,1\right),\prime descend^{\prime }\right)$;22:sortRes(:,2:4)=tmp(idxSort,2:4);


**Algorithm 3** Pseudo code for CountMostUsedOri() function**Input:**  1:Num of pixels: $N_{pix}=N_{ori}\times r\times c$;  2:Sorted magnitude responses of all pixels: $sortRes\in {ℜ}^{N_{pix}\times 4}$.**Output:**  3:Histogram statistics for each candidate orientational filters: hist_count.
  4:
  5:$hist\_count=zeros(N_{ori},1)$;  6:$temp\_resF=zeros(N_{ori},r,c)$;  7:**for** idx = 1 to $N_{pix}$ **do** // for each pixel  8:    currX=sortRes(idx,2);  9:    currY=sortRes(idx,3);10:    currO=sortRes(idx,4);11:    **if** (temp_resF(currO).value(currX,currY)==1) **then**12:        continue;13:    **end if**14:    hist_count(currO)+=1;15:    temp_resF(currO).value(currY,currX)=1;16:**end for**17:hist_count=hist_count./sum(hist_count(:)). // Normalize


### 4.3. Recognition

Following the aforementioned procedures, we have learned the respective feature vectors for each training image through the VF-DCN framework. These feature vectors exhibit versatility, capable of being applied in both classification and verification scenarios.

Under the classification paradigm, the ensemble of feature vectors {$f_{i}$} extracted from the FV ROIs serves as the foundational input for determining the class label (or identity) correlated with each feature vector. To assess the proficiency of VF-DCN in extracting highly discriminative feature vectors, we have opted for a simple yet effective classifier: the *k*-nearest neighbor (*k*-NN) classifier based on Euclidean distance, with *k* = 1 (denoted as 1-NN in the following). This choice is advantageous due to its absence of training requirements and the lack of tunable parameters, ensuring a direct evaluation of the feature vectors’ discriminative power.

Shifting to the verification mode, a crucial matching step ensues. Here, two biometric templates, each encapsulated within their respective feature vectors $f_{i}$ and $f_{j}$, are compared to yield a corresponding distance metric $d_{i,j}=\mathrm{match}\left(f_{i},f_{j}\right)$, where match(·) is the Euclidean distance used for quantitative measure of the similarity between the two feature vectors.

## 5. Experimental Analysis

This section presents the experimental analysis to evaluate the performance of the proposed VF-DCN model. First, Section 5.1 provides the details of the experimental FV databases. Then, Section 5.2 and Section 5.3 present the experimental setting and corresponding evaluation metrics. After that, some key parameters are analyzed in Section 5.4, and the ablation study of the VF-DCN model is presented in Section 5.5. Finally, computational complexity is discussed in Section 5.6, and the comparison with some state-of-the-art methods is presented in Section 5.7.

### 5.1. Experimental Databases

**Figure 7:**
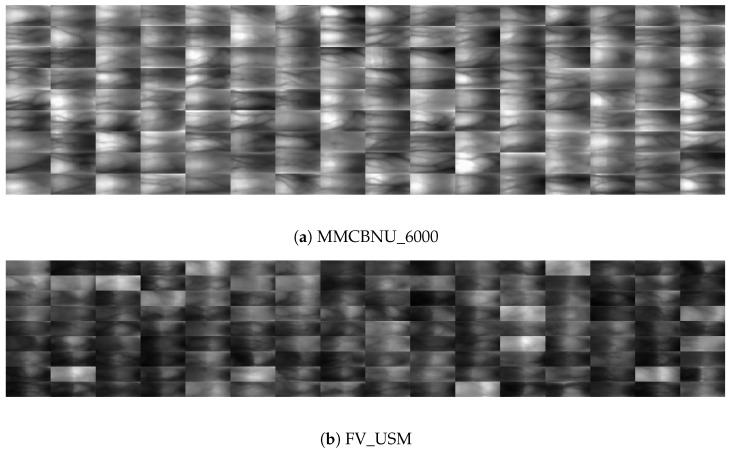

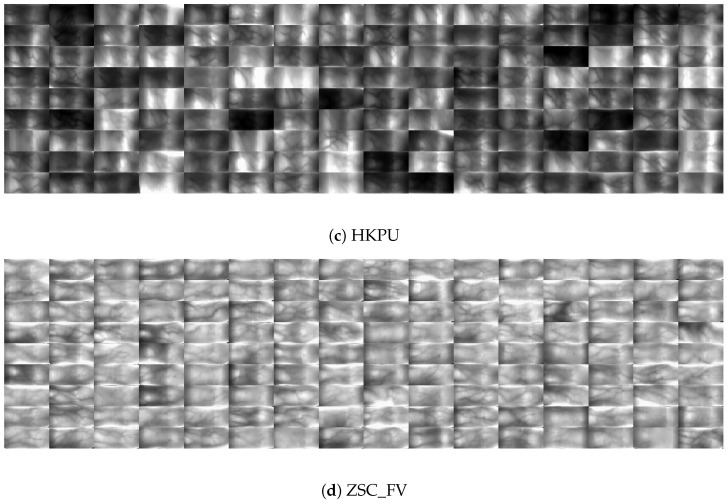
ROI images of four FV databases, in which, ROIs in (**a**,**b**) are provided by the dataset itself, while ROIs in (**c**,**d**) are extracted by 3*σ* criterion [1].

In our experiments, four distinct finger vein databases: MMCBNU_6000 [46], FV_USM [47], HKPU [5], and our Self-Made ZSC_FV [1] are employed to facilitate a fair and comprehensive comparison. These databases capture FV images under diverse conditions and heterogeneous acquisition devices, thereby ensuring the robustness and representativeness of our evaluation for real-world applications. Table 2 shows the pertinent characteristics of the four FV databases, and Figure 7 visually depicts the ROIs of each database.

#### 5.1.1. MMCBNU_6000 [46]

MMCBNU_6000 database (MMCBNU_6000 is available at http://multilab.jbnu.ac.kr/MMCBNU_6000, accessed on 1 December 2023) is created by Jeonbuk National University in Korea. It comprises 6000 FV images from 600 fingers belonging to 100 diverse subjects, encompassing students and professors from CBNU. These subjects originate from 20 countries spanning Asia, Europe, and America, offering a wide range of FV patterns. The database records six fingers per subject—the index, middle, and ring fingers of both hands, with each finger imaged ten times in a single session. The FV images are saved in bitmap (.bmp) format, alongside predefined region of interest (ROI) images with dimensions of 128×60 (as depicted in Figure 7a). Statistical analysis using the 3σ criterion [1] reveals that 94.78% of the images, or 5687 in total, exhibit good quality, while 0.88% (53 images) are of poor quality, with the remainder falling into the medium quality category. This distribution indicates the robustness and suitability of the MMCBNU_6000 database for research and evaluation endeavors.

#### 5.1.2. FV_USM [47]

FV_USM database (FV_USM is available at http://drfendi.com/fv_usm_database/, accessed on 1 December 2023) is created by the University of Sains Malaysia. It comprises 5904 FV images from 492 fingers belonging to 123 individuals, including 83 males and 40 females. These participants, exclusively Asian, are staff and students of USM, spanning ages 20 to 52. For each individual, images of four fingers were captured: the index and middle fingers of both hands. This process was repeated in two distinct sessions, with six captures per finger per session, totaling 12 images per finger. To simulate real-world verification scenarios, where multiple images of the same finger may be available, experimental evaluations often blend images from both sessions for the same finger. All captured FV images are saved in JPEG format, accompanied by predefined ROI with dimensions of 300×100 (as depicted in Figure 7b). Statistical analysis using the 3σ criterion [1] reveals that 83.43% of the images (4926) are of good quality, while 2.98% (176) are deemed poor quality. The remainder falls into the medium-quality category. Although the FV_USM database boasts a slightly lower percentage of top-tier images compared to the MMCBNU_6000 database, it nonetheless offers a valuable resource for research and evaluation purposes.

#### 5.1.3. HKPU [5]

HKPU database (HKPU is available at http://www4.comp.polyu.edu.hk/~csajaykr/fvdatabase.htm, accessed on 1 December 2023), developed by the Hong Kong Polytechnic University, comprises 3132 FV images from 312 fingers of 156 individuals, predominantly under 30 years old. Each participant contributed images of their left index and middle fingers, captured in two separate sessions spanning from one month to over six months apart, with an average interval of 66.8 days. The first session yielded 1872 samples, while the second session gathered 1260 samples from the first 210 fingers. To simulate a real-world scenario, images from the same finger across sessions are intermixed. All finger vein images are saved in bitmap (.bmp) format and were captured under a non-contact acquisition environment, resulting in noise, rotational, and translational variations. The original image size is 513×256 pixels, and undergoes ROI segmentation during preprocessing as described in [1] (refer to Figure 7c). Statistical analysis using the 3σ criterion [1] reveals that 29.31% of the images (918) are classified as good quality, 22.16% (694 images) as poor quality, with the remainder deemed medium quality. This indicates the relatively low proportion of high-quality images in HKPU compared to other databases.

#### 5.1.4. ZSC_FV [1]

ZSC_FV database, created by our team, contains 37,080 FV images collected from 1030 undergraduate students, all within the age range of 18 to 22 years old. Each student contributed 36 images—six samples from the index, middle, and ring fingers of both hands. The acquisition process was conducted indoors under varying illumination conditions, enriching its analytical potential. The capturing device was manufactured by Beijing YanNan Tech Co., Ltd. (Beijing, China). All finger vein images are saved in bitmap (.bmp) format with a resolution of 512×384 pixels. Prior to analysis or use in FVR, these images undergo pre-processing that includes ROI segmentation [1] (as shown in Figure 7d). Statistical analysis using the 3σ criterion [1] reveals that 94.63% (totaling 35,090 samples) comprises good quality images. Conversely, 4.8% of the images (1778 samples) are classified as poor quality, while the remainder falls into the medium quality category. ZSC_FV provides a substantial and diverse dataset of FV images from a young population, and captured under varying conditions, offers compelling experimental results to prove the superiority of our proposed methods.

### 5.2. Experimental Setting

Our experiments were carried out under a computing environment with 3.6 GHz Intel Core i7 CPU (Intel Corporation, Santa Clara, CA, USA) and 32 GB RAM. We adopted an open-set protocol, ensuring that the training and testing sets were entirely non-overlapping. Specifically, for each database, approximately 50% of fingers were randomly selected for training, with the remainder reserved for testing. Notably, in scenarios where a finger was captured across two sessions, we consolidated the images to simulate a realistic data collection scenario, maintaining the distinctiveness between training and testing fingers. The classification and verification tasks were solely executed on the testing set, and the final results were averaged over five iterations for enhanced accuracy. In the verification phase, Euclidean distance served as the metric for similarity assessment.

### 5.3. Evaluation Metrics

As performance metrics, we focused on the equal error rate (EER), accuracy (ACC), and the receiver operating characteristic (ROC) curve, which are widely recognized standards for evaluating the performance of FVR [17].

The EER signifies the optimal balance between the False Acceptance Rate (FAR) and the False Rejection Rate (FRR), with a lower EER indicating superior verification performance. Among these, FAR quantifies the error rate where the unenrolled FV images are accepted as enrolled images, the corresponding formula is shown in Equation (13).

(13) $$\mathrm{FAR}=\frac{Number\;of\;False\;Acceptances}{Number\;of\;Imposter\;Verification\;Attempts}\times 100\%,$$

while FRR represents the error rate where the enrolled FV images are rejected as unenrolled images. The corresponding formula is shown in Equation (14).

(14) $$\mathrm{FRR}=\frac{Number\;of\;False\;Rejections}{Number\;of\;Genuine\;Verifivation\;Attempts}\times 100\%.$$

### 5.4. Key Parameters Analysis

In this experiment, we analyzed some key parameters used in the VF-DCN model, allowing us to understand the specific impact of each parameter on the overall performance. As discussed in Section 3, some key parameters, including the $W_{min}$, *M* (radial scaling factor), and *T* (angular scaling factor) will affect the representation ability of Log-Gabor, so we chose these parameters for testing. When these three parameters are set, the central frequency of the filter $f_{0}$ and the angular standard deviation $\sigma_{\theta }$ are also set by Equations (5) and (8). It should be noted that each sub-experiment focuses on evaluating one parameter while keeping the others fixed according to Table 1, and the FV database adopted is MMCBNU_6000.

By systematically varying each parameter and observing the changes in recognition performance, we can gain insights into how these parameters influence the filter’s effectiveness. Specifically, the diamond convolution structure utilized is [2,7,7,2].

#### 5.4.1. $W_{min}$

**Figure 8:**
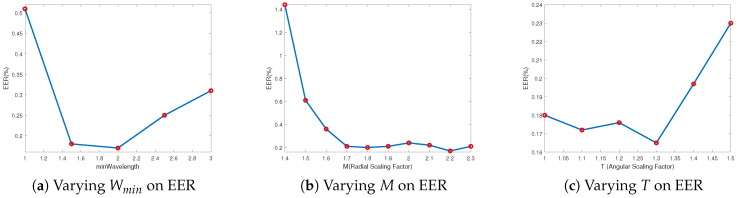
Trend of EER at varying parameters.

This sub-experiment delves into exploring the impact of adjusting $W_{min}$ on recognition performance. Upon setting $W_{min}$, the maximum frequency is derived using Equations (5) and (6). Table 3 presents the recognition performance, and Figure 8a visually illustrates the trend of EER as $W_{min}$ varies. Notably, when the value of $W_{min}$ is set to 2, a relatively superior performance is achieved.

#### 5.4.2. Radial Scaling Factor (*M*)

This sub-experiment investigates the effect of varying the Radial Scaling Factor (*M*) on recognition performance. By adjusting *M*, a sequence of wavelengths and corresponding frequencies are generated, adhering to Equations (5) and (6). Our findings in Table 4 reveal that while variations in *M* have a relatively minor influence on ACC, they significantly impact the EER. Specifically, as *M* increases from 1.4 to 2.2, the EER continuously decreases, indicating an enhanced recognition performance. Figure 8b illustrates the trend of EER, showing how EER improves with increasing *M* values.

#### 5.4.3. Angular Scaling Factor (*T*)

This section investigates the impact of varying *T* (Angular Scaling Factor) on recognition performance. As elaborated in Section 3.3, Equation (8) underscores the role of *T* in influencing the $\sigma_{\theta }$. Table 5 presents the recognition performance under various *T* values. Figure 8c visually depicts the trend of EER as *T* varies. When the value of *T* is set to 1.3, a relatively superior performance is observed, indicating an optimal setting for maximizing recognition accuracy. This adjustment ensures a smooth and effective balance of the angular scaling, thereby enhancing the overall recognition performance.

### 5.5. Ablation Study

In this section, we conduct ablation studies to gain insights into the individual contributions of different scales to the discriminative features and identify the optimal diamond-shaped convolutional structure that maximizes performance. It is important to note that for this study, we utilize the parameter settings detailed in Table 1, specifically *M* = 2.2, $\sigma_{r}$ = 0.55, *T* = 1.3, $W_{min}$ = 2.0, and all ROIs are resized to 32×32.

Firstly, we test the contribution degree of the four scales to the discriminative feature. To do this, we choose 10 orientations from each single scale. In the first column of Table 6, [10,0,0,0] indicates that 10 orientations are chosen from scale S=1, with no orientations selected from the other scales. Similar interpretations apply for [0,10,0,0], which means 10 orientations are chosen from scale S=2, with no orientations selected from the other scales. From Table 6, the EER on S=1 is too high, and the EER on S=4 takes the second place, revealing that using only the smallest (S=1) or largest (S=4) scales results in unacceptably high EERs, akin to the visual blurring that occurs when observing objects at extreme distances or proximities. Conversely, scales at S=2 and S=3 demonstrate relatively lower EERs, suggesting that intermediate scales contribute more effectively to the discriminative features.

**Figure 9:**
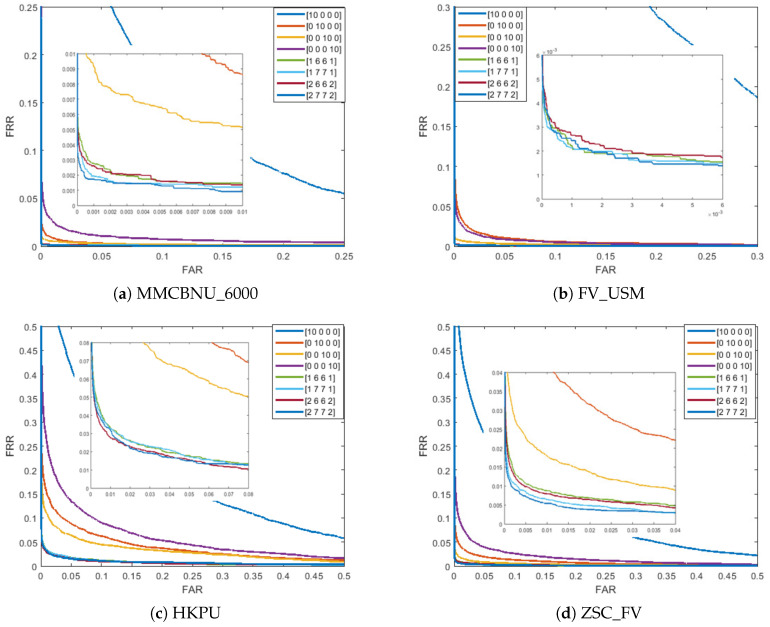
ROC curves of various diamond-shaped convolutional structures on four finger vein databases.

Secondly, we explore the effectiveness of various diamond-shaped convolutional structures. In the first column of Table 6, [2,7,7,2] signifies that the two most predominant orientations are selected on scales S=1 and S=4, respectively, while the seven most predominant orientations are selected on scales S=2 and S=3, respectively. From Table 6, the diamond convolutional structure [2,7,7,2] consistently outperforms other configurations across four databases, as evident from the EER values reported in Table 6 and further illustrated in Figure 9. This optimal structure effectively balances the orientation selection across scales, leading to improved recognition performance.

### 5.6. Feature Extraction Time

In this experiment, we conducted a comprehensive analysis of the feature extraction time for various diamond-shaped convolutional structures. Table 7 presents the feature extraction times (in seconds) for these structures across four FV databases. A clear trend emerges from the results: the fewer orientations selected within a given structure, the lower the time required for feature extraction. Although the structure [2,7,7,2] inevitably takes longer due to its increased number of orientations, it is noteworthy that the time cost for our proposed method remains exceptionally low, at approximately 0.0356 s. This is a testament to the efficiency of our VF-DCN model, even when compared to other DL methods [14], which often come with significantly higher computational overheads. Therefore, our VF-DCN model not only achieves superior performance in terms of recognition accuracy but also maintains an acceptable feature extraction time, making it suitable for real-time applications. The balance between effectiveness and efficiency underscores the practicality and value of our proposed diamond-shaped convolutional structure.

### 5.7. Comparison Experiment

In this experiment, we conducted a thorough comparison of our proposed VF-DCN against the following typical and recent FV feature representation and recognition methods in terms of EER and ACC.

(1) RLF [6]: RLF is a handcrafted method, which combines curvature and radon-like features, can effectively aggregate the dispersed spatial information around the vein structures, thus highlighting vein patterns and suppressing spurious non-boundary responses and noises, obtaining a more smoothing vein structure image. From Table 8, the performance of RLF as a recent handcrafted method is better than GCN and less than other DL methods. It shows that handcrafted methods that are close to human vision also have their advantages.
(2) GCN [36]: GCN (The source code for GCN can be available at https://github.com/jxgu1016/Gabor_CNN_PyTorch, accessed on 12 January 2024) is a Gabor convolutional network with Gabor filters incorporated into DCNNs. The network is composed of four Gabor convolution layers, a Max-pooling and ReLU following the convolution layer, and a dropout layer after the fully connected layer. From Table 8, although as a DL method, the performance of GCN is limited by the depth of the network.
(3) PalmNet [48]: PalmNet (The source code for PalmNet can be available at https://github.com/AngeloUNIMI/PalmNet, accessed on 12 January 2024) is a 3-layer CNN with two Gabor convolutional layers and one binarization layer, which uses an innovative unsupervised training algorithm and can tune filters based on a limited quantity of data. PalmNet is a hybrid method comprised of a Gabor filter and a shallow convolution network. From Table 8, the performance is better than other DL methods, proving the idea that fusing handcrafted and DL is feasible.
(4) SNGR [17]: SNGR was constructed based on a Siamese framework and embedded with a pair of eight-layer tiny ResNets as the backbone branch network. We chose the EER and ACC when the ratio of training and testing data is 9:1, as reported in [17].
(5) SC-SDCN [14]: SC-SDCN is a DL method, which proposes a sparsified densely connected network with separable convolution. The more training data, the better the performance. For comparison fairly, we chose the EER and ACC when the ratio of training and testing data is 5:5. If the training data increase, the performance also improves, which has been reported in [14]. It shows that the DL method is affected by the training data; however, our proposed VF-DCN requires little data.
(6) DenseNet161 [49]: DenseNet161 (The source code for DenseNet161 can be available at https://github.com/ridvansalihkuzu/vein-biometrics, accessed on 12 January 2024) is a DL method. We chose the EER and ACC when the ratio of training and testing data is 9:1, which has been reported in [17].

Despite the unique strengths exhibited by all the methods under consideration, the proposed VF-DCN model demonstrates superior performance across four distinct databases, as shown in Table 8. Our method achieves low EERs of 0.17%, 0.19%, 2.11%, and 0.65%, and high ACCs of 100%, 99.97%, 98.92%, and 99.36% on the MMCBNU_6000, FV_USM, HKPU, and ZSC_FV databases, respectively. This achievement validates the feasibility of our innovative approach, which integrates simulated retinal imaging techniques with a combination of Log-Gabor filters and a diamond-shaped convolutional structure. The successful integration of these components not only enhances the network’s ability to capture intricate FV features but also showcases the potential of this novel approach in advancing the field of FV technology.

**Table 8:** Comparison with other methods on four FV databases.

Methods	MMCBNU_6000		FV_USM		HKPU		ZSC_FV	
	**EER**	**ACC**	**EER**	**ACC**	**EER**	**ACC**	**EER**	**ACC**
RLF [6]	0.78%	-	0.87%	-	2.49%	-	1.39%	-
GCN [36]	1.86%	98.74%	2.05%	98.72%	-	-	-	-
PalmNet [48]	0.21%	99.97%	0.28%	99.97%	2.73%	99.30%	0.73%	99.10%
SNGR [17]	0.52%	99.55%	0.5%	99.74%	-	-	-	-
SC-SDCN [14]	0.58%	99.68%	0.82%	99.62%	-	-	-	-
DenseNet161 [49]	0.60%	99.57%	1.48%	98.94%	-	-	-	-
**VF-DCN**	0.17%	100%	0.19%	99.97%	2.11%	98.92%	0.65%	99.36%

## 6. Conclusions

In this paper, we carried out a hybrid exploration of Log-Gabor and a diamond convolutional structure. The advantages of the proposed VF-DCN are as follows:

(1) IntegrationofLog-GaborFilters: Log-Gabor filters are well-suited for natural image processing due to their ability to capture the statistical properties of natural scenes. By incorporating Log-Gabor filters into our network architecture, we effectively leverage their benefits for improved image feature extraction and representation.
(2) DiamondConvolutionalStructure: This structure enables the network to capture spatial information in a more efficient and effective manner, leading to improved performance.
(3) SimulatingRetinalImaging: By combining Log-Gabor filters and diamond convolutions, we created a network that simulates the processes of the human retina. This approach results in a network that is better able to represent and process visual information in a way that is similar to the human visual system.
(4) ImprovedPerformance: The fact that VF-DCN achieves the best performance compared to other methods is a clear indication that our approach is effective. This not only validates our idea but also demonstrates the potential of combining Log-Gabor filters and diamond convolutions for visual information processing tasks.
(5) PotentialforFurtherApplications: The success of VF-DCN in achieving superior performance suggests that this approach has the potential to be applied to a wide range of image processing and computer vision tasks, such as object detection, image segmentation, and visual recognition.

While the VF-DCN excels as an efficient lightweight network model, featuring just two convolutional layers, it is prudent to acknowledge its inherent limitations in extracting deeper, more abstract features. Consequently, there is a pressing need to delve deeper into extending this network model, exploring ways to transform it into a deeper, more comprehensive architecture. Furthermore, although the adaptive learning strategy of orientational filters is indeed inspired by the intricate workings of the human visual system, it is imperative to undertake rigorous research to determine the optimal number of orientational filters at each scale. Looking ahead, we plan to continue this line of research and endeavor to integrate VF-DCN with self-attention mechanisms, thereby enhancing the network’s ability to mimic the fundamental principles underlying biological visual imaging systems even more closely.

## Figures and Tables

**Table 1:** Parameters setting of the 2D Log-Gabor filter.

	Parameter	Value	Description
Radial	$W_{min}$	2	Wavelength of the smallest scale filter
	$\sigma_{r}$	0.55	Radial standard deviation
	$N_{scale}$	4	Number of radial filter scales
	*M*	2.2	Radial scaling factor
Angular	$\sigma_{\theta }$	–	Angular standard deviation
	$N_{ori}$	10	Number of filter orientations
	*T*	1.3	Angular scaling factor

**Table 2:** Details of four FV databases (For column ‘Fingers’, i: index, m: middle, r: ring. For column ‘Hands’, l: left hand, r: right hand).

Databases	Total Num of Images	Num of Finger Classes	Num of Subjects	Fingers	Hands	Num of Images per Finger	Sessions	ROI
MMCBNU_6000	6000	600	100	i, m, r	l, r	10	1	provided
FV_USM	5904	492	123	i, m	l, r	12	2	provided
HKPU	3132	312	156	i, m	l	6/12	2	3σ criterion [1]
ZSC_FV	37,080	6189	1030	i, m, r	l, r	6	1	3σ criterion [1]

**Table 3:** Varying $W_{min}$ results on recognition performance.

	1.0	1.5	**2.0**	2.5	3.0
EER	0.51%	0.18%	0.17%	0.25%	0.31%
ACC	99.97%	100%	100%	99.97%	99.93%

**Table 4:** Different *M* (radial scaling factor) results on recognition performance.

	1.4	1.5	1.6	1.7	1.8	1.9	2.0	2.1	**2.2**	2.3
EER	1.44%	0.61%	0.36%	0.21%	0.20%	0.21%	0.24%	0.22%	0.17%	0.21%
ACC	99.31%	99.84%	99.97%	100%	100%	100%	100%	99.97%	100%	100%

**Table 5:** Different *T* (angular scaling factor) results on recognition performance.

	1.0	1.1	1.2	1.3	1.4	1.5
EER	0.180%	0.172%	0.176%	0.165%	0.197%	0.230%
ACC	100%	100%	100%	100%	100%	100%

**Table 6:** Number of convolution results on four FV databases.

Number of Convolution	MMCBNU_6000		FV_USM		HKPU		ZSC_FV	
	**EER**	**ACC**	**EER**	**ACC**	**EER**	**ACC**	**EER**	**ACC**
[10,0,0,0]	12.88%	46.37%	24.34%	19.93%	19.45%	53.11%	14.11%	37.76%
[0,10,0,0]	0.91%	99.84%	1.95%	98.81%	7.33%	94.99%	2.68%	93.15%
[0,0,10,0]	0.60%	99.85%	0.71%	99.80%	5.91%	95.50%	1.55%	97.48%
[0,0,0,10]	1.89%	99.34%	1.70%	99.25%	9.38%	88.40%	4.30%	83.13%
[1,6,6,1]	0.21%	100%	0.19%	99.97%	2.41%	98.67%	0.91%	98.74%
[2,6,6,2]	0.21%	100%	0.21%	99.97%	2.20%	98.92%	0.83%	98.80%
[1,7,7,1]	0.18%	100%	0.20%	99.97%	2.44%	98.61%	0.72%	99.18%
[2,7,7,2]	0.17%	100%	0.19%	99.97%	2.11%	98.92%	0.65%	99.36%

**Table 7:** Feature extraction time(/s) of various diamond structures on four FV databases.

Diamond-Shape	MMCBNU_6000	FV_USM	HKPU	ZSC_FV
[10,0,0,0]	0.0030	0.0031	0.0024	0.0059
[0,10,0,0]	0.0030	0.0031	0.0023	0.0058
[0,0,10,0]	0.0025	0.0026	0.0019	0.0043
[0,0,0,10]	0.0019	0.0019	0.0017	0.0030
[1,6,6,1]	0.0068	0.0066	0.0273	0.0117
[2,6,6,2]	0.0111	0.0103	0.0272	0.0386
[1,7,7,1]	0.0111	0.0109	0.0282	0.0178
[2,7,7,2]	0.0356	0.0344	0.0285	0.0408

## Data Availability

The original contributions presented in the study are included in the article, further inquiries can be directed to the corresponding authors.
